# 1261. Do All Patients Prefer Telehealth Care?

**DOI:** 10.1093/ofid/ofac492.1092

**Published:** 2022-12-15

**Authors:** Smitha Gudipati, Monica Lee, Joanne Huitsing, Norman Markowitz, Indira Brar

**Affiliations:** Henry Ford Health System, Detroit, Michigan; Henry Ford Health System, Detroit, Michigan; Henry Ford Hospital, detroit, Michigan; Henry Ford Health System, Detroit, Michigan; Henry Ford Hospital, detroit, Michigan

## Abstract

**Background:**

COVID-19 has threatened health care for many individuals. Restriction of resources, redeployment of staff, and patient reluctance to make clinic appointments disrupts continuity of care of existing patients and limits access to care of new ones. To overcome this, our HIV clinic aggressively promoted a telehealth, MyChart (MC) application, and provided smart phone technology to those in need. Despite these efforts, we found that utilization of telehealth accounted for 4.7% of HIV clinic visits, compared to 25% in internal medicine clinics. In this report, we sought to obtain reasons why our patients were reluctant to use telehealth even in the midst of a pandemic.

**Methods:**

All Ryan White (RW) people living with HIV (PLWH) at Henry Ford Hospital that were initiated in our telehealth pilot program were surveyed on the underutilization of MC. Utilization was determined by if PLWH responded to a MC notification sent by the telehealth navigator. Activity level was established on MC (Figure 1), and if PLWH did not respond, they were called as a follow up for survey answers.

**Figure:**
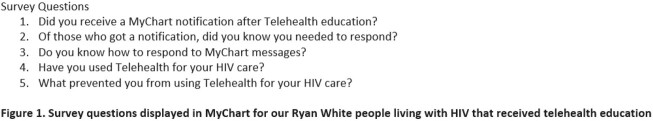

**Results:**

From 10/2020 – 01/2022, 206 PLWH were enrolled into our pilot program and given telehealth education. Of those successfully enrolled: 83.7% were black, 73% male, 57% were older than 45 years, 88% lived in Wayne County, and 27 needed and received pre-loaded smart phones. When contacted, 90 (44%) interacted on MC, 61 (29%) were unable to be reached and 55 (27%) successfully completed the survey (Figure 2). When asked why telehealth was not utilized, 27 (49%) stated they preferred in-person visits (Figure 3).

**Figure:**
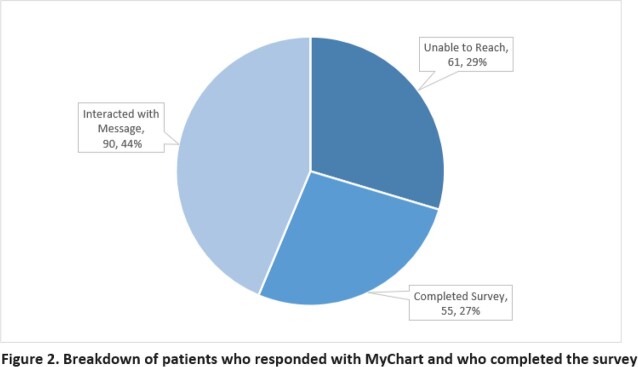

**Figure:**
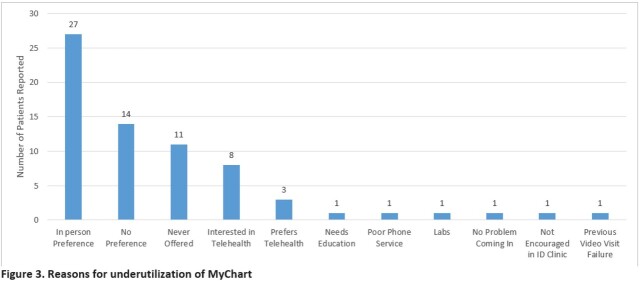

**Conclusion:**

Telehealth programs can help overcome barriers to HIV care and maintain patient engagement when crises interrupt traditional care models. However, our study suggests that our PLWH preferred and felt safe engaging with in-person visits despite telehealth education and smartphone supplementation even in a pandemic. As the future of medicine moves towards telehealth management, we must not forget our vulnerable populations and find opportunities to safely engage with in-person visits.

**Disclosures:**

**Indira Brar, MD**, Gilead: Grant/Research Support|Gilead: speakers bureau|Janssen: Grant/Research Support|Janssen: speakers bureau.

